# Parasitic infections during pregnancy need not affect infant antibody responses to early vaccination against *Streptococcus pneumoniae*, diphtheria, or *Haemophilus influenzae* type B

**DOI:** 10.1371/journal.pntd.0007172

**Published:** 2019-02-28

**Authors:** Noah D. McKittrick, Indu J. Malhotra, David M. Vu, Derek B. Boothroyd, Justin Lee, Amy R. Krystosik, Francis M. Mutuku, Charles H. King, A. Desirée LaBeaud

**Affiliations:** 1 Division of Infectious Diseases, Department of Medicine, Stanford University School of Medicine, Stanford, California, United States of America; 2 Center for Global Health and Diseases, Case Western Reserve University School of Medicine, Cleveland, Ohio, United States of America; 3 Division of Infectious Diseases, Department of Pediatrics, Lucille Packard Children’s Hospital at Stanford School of Medicine, Stanford, California, United States of America; 4 Quantitative Sciences Unit, Department of Medicine, Stanford University School of Medicine, Stanford, California, United States of America; 5 Department of Environment and Health Sciences, Technical University of Mombasa, Mombasa, Kenya; Centers for Disease Control and Prevention, UNITED STATES

## Abstract

**Background:**

Globally, vaccine-preventable diseases remain a significant cause of early childhood mortality despite concerted efforts to improve vaccine coverage. One reason for impaired protection may be the influence of prenatal exposure to parasitic antigens on the developing immune system. Prior research had shown a decrease in infant vaccine response after *in utero* parasite exposure among a maternal cohort without aggressive preventive treatment. This study investigated the effect of maternal parasitic infections on infant vaccination in a more recent setting of active anti-parasitic therapy.

**Methodology/Principal findings:**

From 2013–2015, 576 Kenyan women were tested in pregnancy for malaria, soil-transmitted helminths, filaria, and *S*. *haematobium*, with both acute and prophylactic antiparasitic therapies given. After birth, 567 infants received 10-valent *S*. *pneumoniae* conjugate vaccine and pentavalent vaccine for hepatitis B, pertussis, tetanus, *H*. *influenzae* type B (Hib) and *C*. *diphtheriae* toxoid (Dp-t) at 6, 10, and 14 weeks. Infant serum samples from birth, 10 and 14 weeks, and every six months until age three years, were analyzed using a multiplex bead assay to quantify IgG for Hib, Dp-t, and the ten pneumococcal serotypes. Antenatal parasitic prevalence was high; 461 women (80%) had at least one and 252 (43.6%) had two or more infections during their pregnancy, with the most common being malaria (44.6%), *S*. *haematobium* (43.9%), and hookworm (29.2%). Mixed models comparing influence of infection on antibody concentration revealed no effect of prenatal infection status for most vaccine outcomes. Prevalences of protective antibody concentrations after vaccination were similar among the prenatal exposure groups.

**Conclusions/Significance:**

These findings are in contrast with results from our prior cohort study performed when preventive anti-parasite treatment was less frequently given. The results suggest that the treatment of maternal infections in pregnancy may be able to moderate the previously observed effect of antenatal maternal infections on infant vaccine responses.

## Author summary

This mother-baby cohort study continued our investigations into the potential impact of a mother’s parasitic infection(s) during pregnancy on a baby’s ability to respond to early life vaccinations. In a rural Kenyan setting where malaria and helminth infections are common, we tested infants’ anti-vaccine antibody responses over time for up to three years of age after early vaccination against *Streptococcus pneumoniae* (the pneumococcus), diphtheria, and *Haemophilus influenzae* B (Hib). In contrast to the results for our previous 2006–2009 cohort, for whom antenatal parasite exposure reduced responses to diphtheria and Hib, our more recent 2013–2015 cohort did not show consistent evidence of an effect of antenatal maternal infection on subsequent infant vaccine responses. We conclude that the impact of antenatal infections on infant immune response can be mitigated, and that present-day screening and preventive therapies during pregnancy may have achieved this effect.

## Introduction

The global burden of vaccine-preventable diseases remains high, especially among children under 5 years old, with 1 to 2 million deaths recorded annually [1]. The encapsulated bacteria *Streptococcus pneumoniae* and *Haemophilus influenzae* type B (Hib) cause the majority of child deaths from pneumonia, of which >99% occur in less-developed countries [2]. Widespread anti-pneumococcus and anti-Hib vaccination programs have been implemented by the WHO’s Expanded Program on Immunization, with reductions in rates of vaccine-targeted pneumonias and of carriage of vaccine-covered serotypes [3]. However, these successes often fail to highlight the fact that children in developing nations often have less robust responses to vaccines. This has been observed in several campaigns, for example programs administering Bacille Calmette-Guérin (BCG) for tuberculosis prevention [4], and those administering typhoid [5] and measles vaccinations [6]. A recent polio outbreak in Africa highlighted this problem, wherein a Nigerian polio epidemic spread to Ghana, Botswana, and Kenya, despite > 90% vaccination rates in those nations [7].

Many factors are likely involved in apparent reductions in vaccine efficacy, including nutritional deficits, cold chain problems, and incomplete coverage and/or uptake within mass immunization campaigns. However, a growing body of evidence supports the hypothesis that chronic parasitic infections may also influence response to vaccination. Intestinal helminth infections have decreased immunization efficacy in animal models [8], and in human studies, BCG and tetanus vaccine antibody responses have been found to be diminished in the setting of *Schistosoma* infections [9, 10]. In addition, childhood responses to tetanus, Hib, and typhoid vaccination appear to be attenuated by malaria infection [11–13].

While the immunomodulatory effects of parasites have been extensively studied, the host-parasite relationship and its fetal effects during pregnancy are poorly understood. Our group has observed that chronic maternal parasitic infections can influence the developing immunity of the child *in utero*. Transplacental shift of parasite antigens exposes the fetus to materials that evoke immunomodulatory effects such that parasite-specific B- and T-cells are already present at birth [14]. In our previous 2006–2009 study, such parasite exposure was associated with a decreased response to Hib and diphtheria immunization in early infancy [15, 16]. These effects appeared to be due to an “imprinting” phenomenon that positively or negatively skewed neonatal immune response to parasite-specific antigens [17], and in turn, may have had a bystander effect that impaired infant responses to unrelated antigens such as those found in early childhood vaccines [18].

In many countries, endemic parasitic infections remain a significant public health challenge. Prenatal screening and treatment for these infections is becoming standard for antenatal care based on WHO guidelines, but maternal parasitic infections continue to occur and/or relapse at significant rates. While vaccination against encapsulated bacteria is also becoming the norm in the developing world, there is potential that this effort may be hindered by prevalent parasite exposures. In our previous study of a 2006–2009 mother infant cohort, we found maternal infections during pregnancy to be associated with reduced infant responses to diphtheria toxoid and *H*. *influenzae* type B polyribitol phosphate (PRP) [15, 16]. In the present 2013–2015 study, we revisited the effects of a mother’s prenatal parasitic infections on her infant’s response to early childhood vaccination, specifically, against the previously affected diphtheria and Hib, and in the wake of the introduction of 10-valent pneumococcal conjugate vaccine in the Kenyan schedule [18], against *Streptococcus pneumoniae* serotype vaccine antigens 1, 4, 5, 6B, 7F, 9V, 14, 18C, 19F and 23F.

## Methods

### Ethics statement

Ethical approval of this study protocol was obtained from the Kenyatta National Hospital Ethical Review Committee (protocol #P85/03/2013) and from the Institutional Review Boards at Case Western Reserve University (IRB #01-13-13) and Stanford University School of Medicine (protocol #IRB-31468). Participating mothers provided written informed consent for their own participation and that of their infants.

### Study design and participants

In this prospective cohort study, pregnant women were enrolled at the Msambweni County Referral Hospital antenatal clinic in Msambweni, Kenya, a predominantly rural area on the southern coast with high co-prevalence of parasitic diseases [19, 20]. Enrollment occurred between July 2013 and July 2015. Mothers were followed until delivery, and their newborn infants were subsequently followed until up to three years of age.

Inclusion and exclusion criteria and censoring events for the cohort study are listed below:

*Inclusion criteria*:

- Mother at least 15 years of age
- Willing to provide informed consent
- Current pregnancy
- Apparent good health
- Long-term resident in Msambweni locations who anticipated residing in the area during the study period (at least 3 years)
- Willingness to donate blood (peripheral venous blood or finger stick blood as per the protocol) during ANC visits and at the time of delivery
- Willingness to share with the research team, on a confidential basis, her human immunodeficiency virus (HIV) testing results from the ANC-linked voluntary counseling and treatment (VCT) program
- Willingness of the infant’s mother to participate in prenatal and postnatal care at Msambweni District Hospital
- Willingness of the mother/caregivers to participate in a prospective survey that involves bi-annual venipuncture (3–5 mL blood volume) of the infant commencing at 6 months (plus or minus 2 months of age) and ending at age 36 months
- Multiple births could be included

[Note: Pregnant women were allowed to enroll irrespective of their gestational age, although they could not enroll at delivery because we could not assure adequately informed consent. However, potential participants were strongly encouraged to come to the clinic for prenatal care early in the second trimester (ideally <16 weeks gestation) both to ensure adequate prenatal care for the mother and unborn infant and to provide sufficient follow-up time to collect antenatal study samples from the mother. Maternal cohort participation in antenatal clinic care ranged from 1 week to 35 weeks; the median was 15 weeks (interquartile range 11–19 weeks).]

*Exclusion criteria*:

- Preterm delivery less than 34 weeks gestation
- Failure to deliver in the hospital
- Evidence of placenta previa
- Maternal chorioamnionitis
- Receipt of immunosuppressive drugs during pregnancy
- Hemoglobin <6.07 g/dL for mother
- 1+ or greater glucose score on urine dipstick examination
- Administration of immunoglobulins and/or any blood products within the 3 months preceding enrollment in the study
- Current active participation in a separate interventional treatment study
- Acute or chronic pulmonary, cardiovascular, hepatic, renal or neurological condition or any other findings that in the opinion of the Clinical Officer or Principal Investigator might have increased the risk of participating in the study
- Other conditions that would jeopardize the safety or rights of a participant in the trial or would render the participant unable to comply with the protocol.

*Censoring events for the mother and newborn (and*, *by default*, *the infant’s mother)*:

- Mother delivered elsewhere, migrated out of area, died, or experienced a spontaneous abortion or stillbirth
- APGAR score for the infant was less than 5 at 10 minutes after birth
- Meconium aspiration, respiratory distress syndrome, or condition requiring neonatal resuscitation
- Extremely low birth weight (less than 1500 grams)
- Refusal to continue to participate in the study

### Procedures

Maternal participants were screened at each prenatal visit and delivery for malaria (by blood smear and DNA PCR), for *S*. *haematobium* (by urine filtration egg counts and plasma anti-soluble worm adult protein [SWAP] IgG4) [21], for intestinal helminths (by quantitative stool microscopy using the Kato-Katz method [22]), and for lymphatic filariasis (by detection of circulating serum filarial Og4C3 antigen) [23]. Mothers received intermittent preventive treatment for malaria in pregnancy (IPTp) with sulfadoxine-pyrimethamine at each monthly clinic visit, and were actively treated for any symptomatic intercurrent malaria with artemether-lumefantrine. Upon clinic enrollment, all mothers were dispensed an initial ‘preventive’ dose of mebendazole (500 mg). Any participants who tested positive for intestinal helminths received additional single 500 mg dosing with mebendazole within 72 hours. In accordance with Kenyan Ministry of Health guidelines in place during the period of the study, pregnant mothers were not given treatment for schistosomiasis or filariasis during the antenatal period.

Blood, urine, and stool samples were collected from mothers and infants at all study visits, as well as infant cord blood. On delivery, infant anthropometrics and temperature were recorded. APGAR score was assessed at 1, 5, and 10 minutes, and gestational age was estimated by dates and by the revised Dubowitz clinical measurement [24], as prenatal ultrasound examination was not available. Infants received standard immunizations per the Kenya Ministry of Health guidelines, including the pentavalent diphtheria-tetanus-whole cell pertussis-hepatitis B-Hib vaccine, and ten-valent pneumococcal-conjugate vaccine for serotypes 1, 4, 5, 6B, 7F, 9V, 14, 18C, 19F, and 23F (Synflorix, GSK) at 6, 10, and 14 weeks of age.

Serum antibody levels to *S*. *pneumoniae* serotype antigens, Hib PRP, and diphtheria CRM197 toxoid were measured using a fluorescent multiplexed bead-based immunoassay (Luminex, Austin, TX) as previously described [25]. The twelve vaccine antigens were coupled to carboxylated microspheres and then incubated with patient serum. Beads with subject antibodies bound to the antigen were then quantified on a BioPlex MAGPIX multiplex reader (BioRad, Hercules, CA).

A stock serum (composed of 007SP (anti-pneumococcal polysaccharide (PnPs) human serum [26] and 09/222 (anti-Hib human serum, NIBSC, UK) was used to create a standard curve for each assay run. IgG levels against the vaccine antigens were then determined by interpolation of the fluorescent signal observed for the subject sample within the standard curve of the stock serum.

### Statistical analysis

Results were analyzed using R statistical software (R Core Team, 2016). Based on mothers’ prenatal infection history, three classes of infants were compared: i) those of “uninfected” mothers, which was defined as women who had no evidence of any parasitic disease at any visit; ii) those of “infected” mothers who had one or more infections at any time; and iii) those of mothers who had “two or more infections,” which meant having evidence of two or more concurrent infections at any one time before birth. Baseline characteristics were compared via unpaired t-tests, Pearson’s Chi-square testing or Fisher’s Exact test, as appropriate. Individual parasitic infections considered in the analysis included malaria, *S*. *haematobium*, hookworm, and/or filaria, as well as a category of “any soil-transmitted helminth” (i.e., positive either for hookworm, *Trichuris trichiura*, *Ascaris lumbricoides*, or *Strongyloides stercoralis*).

A linear mixed effects model was used to compare the effect of each infection, whether at any prenatal visit, at delivery, or at either time, on the trajectory of antibody concentration against each of the studied vaccine antigens. Antibody concentrations were log-transformed to achieve approximately normal distributions then a model was constructed for each antigen response with fixed effects for infection, gender, and visit number, as well as a random effect for unique subject identification number. Infection variables were considered having a significant main effect on the model prediction if they had a *P* value ≤ 0.05. A second model was then constructed to examine the interaction between infection status and child age at the time of visit in predicting antibody concentration, using a fixed effect for interaction of infection and time point, along with the previously included variables. In this model, an interaction at a particular time point was considered statistically significant at *P* ≤ 0.05. Correction for multiple comparisons was not included in this analysis.

We determined the percentage of infants having protective antibody levels at birth, 6 months, and 24 months by enumerating the tested subjects who had antibody levels above threshold concentrations previously established as providing protection against invasive bacterial infections at each time point. The cutoff values were: ≥ 0.35 μg/ml for pneumococcal serotypes, ≥ 0.1 IU/ml for diphtheria, and ≥ 1.0 μg/ml for Hib [27].

## Results

### Enrollment and baseline characteristics

**Fig 1:**
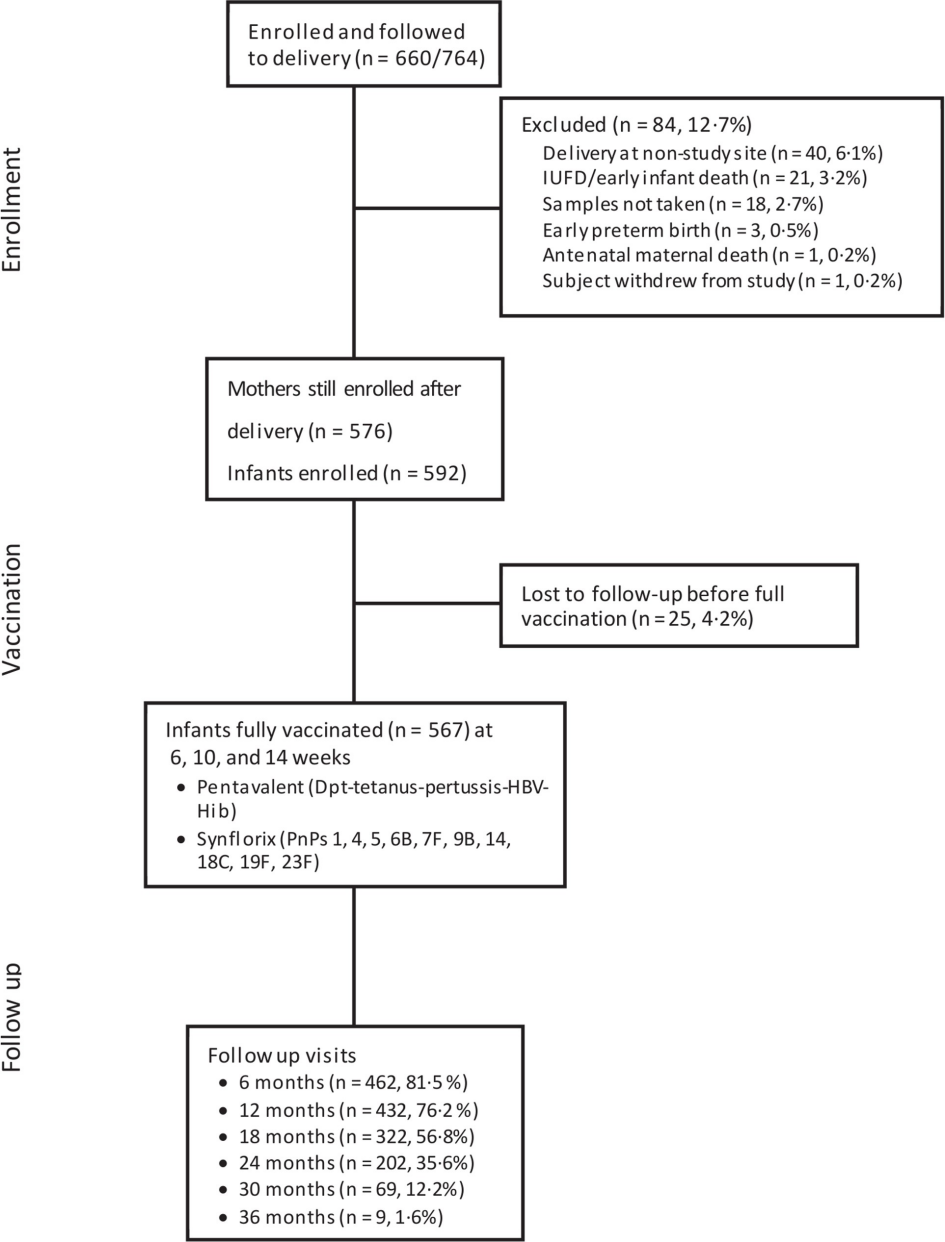
Study design and enrollment numbers. The flow diagram indicates maternal and infant participation at the stages of study enrollment, delivery, vaccination, and biannual infant follow-up.

From July 2013 through July 2015, a total of 764 mothers were enrolled during prenatal visits, with 660 followed to delivery (Fig 1). Mean estimated gestational age (by last menstrual cycle) at the first prenatal visit was 23.1 weeks (95% CI 22.7–23.5). During the course of their prenatal care, 686/764 (90%) of mothers received at least one dose of sulfadoxine-pyrimethamine as IPTp, and 709 (93%) received mebendazole for STH infections. Sixty-six mothers (8.6%) received artemether-lumefantrine treatment for acute malaria during their pregnancy.

After delivery, 576 mothers remained enrolled in the study; the 84 drop-outs were due to: i) delivery off-site (n = 40, 6.1%), ii) failure to collect samples at delivery (n = 18, 2.7%), iii) stillbirth or early infant death (n = 21, 3.2%), iv) antenatal maternal death (n = 1, 0.2%), v) preterm birth (n = 3, 0.5%), or vi) subject withdrawal from study (n = 1, 0.2%) (Fig 1). Among these 576 mothers, 566 (98%) had received antenatal IPTp antimalarial prophylaxis with sulfadoxine-pyrimethamine. As IPTp was dispensed at each ANC visit, the total number of doses taken during pregnancy varied with mother’s clinic participation. The median number of doses given was three (range = 0 to 7 doses). Sixty-six mothers (11.4%) had been given artemether-lumefantrine at some point during pregnancy for symptoms of acute malaria. Eighty percent of mothers with detectable malaria parasitemia (N = 231) cleared their parasitemia with therapy. Additionally, 555 of the mothers (96%) received one or more doses of prenatal mebendazole, and 133 (23%) had received two or more supplemental mebendazole doses due to interval positive stool egg detection. Overall, 116/198 (59%) of women with detectable stool STH cleared their infections with therapy during their pregnancy.

There were 592 infants born to the 576 cohort mothers, due to 16 twin births. Twenty-five infants (4.2%) were lost to follow-up before the first immunization; continuing cohort infant participants were followed for a mean of 17.6 months (SD 8.2, range 1–37). In the data presented below, cord blood antibody levels of the newborns subsequently lost to follow up were included in the determination of the prevalence of protective levels of antibodies at birth.

Baseline characteristics for the mothers fully tested for infection and their infants are shown in Table 1. Uninfected mothers were, on average, 1.9 years older than infected mothers, and 2.0 years older than mothers having two or more infections, but these differences were not statistically significant. Uninfected women also had a higher average body mass index [BMI] compared to infected mothers or polyparasitized mothers, but these differences also did not reach statistical significance. Monthly household expenditures, bed net use, HIV prevalence, primigravid status, maternal parity, and delivery hemoglobin were not significantly different among the groups. However, none of the parasite-uninfected mothers was HIV positive (compared to 5.6% of parasite-infected mothers), and fewer of the parasite-uninfected mothers were in their first pregnancy (14%) as compared to infected mothers (24%). There were no significant differences between these groups for all infant characteristics measured at birth, including sex distribution, birth weight, birth length, APGAR scores, estimated gestational age (Dubowitz score), or rate of preterm births.

**Table 1:** Baseline characteristics of the mother-infant cohort enrolled 2012–2015 ^a^.

Maternal characterisitics	Uninfected mother (N = 36)	Mother infected (N = 461)	*P* value^b^	Mother with ≥ 2 infections (N = 252)	*P* value^b^
Age	28.0 (6.6)	26.1 (6.3)	0.083	26 (6.5)	0.086
Household expenditures^c^	4.5 (0.8)	4.5 (0.7)	0.999	4.4 (0.7)	0.432
Used bed net	34 (94%)	435 (94%)	0.983	230 (91%)	0.519
HIV positive	0 (0%)	26 (5.6%)	0.282	11 (4.4%)	0.576
Primigravid	5 (14%)	110 (24%)	0.172	63 (25%)	0.142
Parity	2.6 (1.8)	2.1 (1.8)	0.109	2.1 (1.9)	0.138
Body mass index	25 (4.5)	24 (4.3)	0.181	24 (4.0)	0.168
Delivery hemoglobin (gm/dL)	9.5 (2.1)	9.7 (2.1)	0.582	9.7 (2.3)	0.622
**Infant characteristics**	**(N = 36)**	**(N = 473)**		**(N = 258)**	
Female	18 (50%)	255 (54%)	0.650	144 (56%)	0.511
Birth weight (kg)	3.04 (0.47)	3.00 (0.44)	0.601	3.02 (0.44)	0.801
Birth length (cm)	49.2 (2.1)	48.8 (2.1)	0.272	48.9 (2.0)	0.404
Head circumference (cm)	33.9 (1.2)	33.6 (1.5)	0.242	33.7 (1.4)	0.416
APGAR score 1 minute	9.3 (0.7)	9.2 (1.0)	0.556	9.2 (0.9)	0.523
APGAR score 5 minutes	9.8 (0.4)	9.7 (0.7)	0.398	9.8 (0.6)	0.999
APGAR score 10 minutes	10 (0)	9.9 (0.6)	0.318	9.9 (0.6)	0.318
Dubowitz score^d^	37.7 (1.2)	37.9 (1.6)	0.463	38 (1.6)	0.280
Preterm delivery	8 (22%)	92 (20%)	0.687	51 (20%)	0.730

^a^ Data presented as mean (SD), or as n (%). Because of twin deliveries, there are more infants than mothers in some categories.

^b^ P value for differences between left-flanking category value and that for the uninfected mother category, as determined by unpaired t-test or chi-square testing.

^c^ Estimated monthly expenses in thousands of Kenya shillings per month.

^d^ Estimated gestational age based on 34 physical findings and neurological assessments at birth.

### Parasitic infection prevalence

Parasitic infections were common in the maternal cohort (see Table 2). A total of 433/576 (75%) of mothers of participating infants had had at least one parasitic infection during pregnancy, and 249 (43%) of the mothers were infected at delivery. Only 36 women (7.2%) who completed all testing had no evidence of infection by any of the eight parasites tested for in our study. The most common maternal infection was malaria, with 231 (40%) of mothers found to be infected at antenatal visits, 49 (8.5%) at delivery, and 257 (45%) at any time point of surveillance. The mothers’ next most common infection was *S*. *haematobium* with 253 (44%) found to be infected. There were 168 hookworm infections (29%), and 132 women were filaria-infected (23%). Soil-transmitted helminths, as a class, were also quite frequent, with 34% of women having evidence of at least one intestinal helminth infection. During prenatal testing, 233 mothers (40%) tested positive for more than one parasite and 66 mothers (11%) had more than one infection at the time of delivery. Unfortunately, a significant fraction of mothers did not provide stool (n = 200/576, 35%), urine (n = 176, 31%), or have *S*. *haematobium* or *W*. *bancrofti* blood testing (n = 136, 24%) completed at delivery. Therefore, helminth parasite prevalence at delivery for these infections was calculated only for the group with non-missing data. In order to avoid any misclassification, mothers were considered as “uninfected” only if they had all parasite testing completed and were negative for all pathogens. As a result, 79 women who were negative only on partial testing were formally excluded from the “uninfected” group included in our analysis.

**Table 2:** Maternal prevalence of parasite infections during pregnancy.

Infection	During antenatal care^a^	At delivery^b^	At either time^a^
Hookworm	154 (27%)	33 (9%)	168 (29%)
*Trichuris trichiura*	62 (11%)	13 (3%)	67 (12%)
*Ascaris lumbricoides*	10 (2%)	1 (0.3%)	10 (2%)
*Strongyloides stercoralis*	17 (3%)	2 (0.5%)	19 (3%)
Any STH	198 (34%)	41 (11%)	212 (37%)
*Giardia lamblia*	6 (1%)	3 (1%)	9 (2%)
*Schistosoma haematobium*	191 (33%)	167 (31%)	253 (44%)
*Wuchereria bancrofti*	117 (20%)	62 (14%)	132 (23%)
Malaria	231 (40%)	49 (8.5%)	257 (45%)
Any parasite	433 (75%)	249 (43%)	461 (80%)
Polyparasitic	233 (40%)	66 (11%)	276 (48%)
2 infections	144 (25%)	53 (9%)	177 (31%)
3 infections	60 (10%)	11 (2%)	68 (12%)
4 infections	25 (4%)	2 (0.3%)	27 (5%)
5 infections	4 (1%)	0 (0%)	4 (1%)

^a^ Data presented as n (%) for the total enrolled maternal group (N = 576).

^b^ Not all samples were obtained at delivery. Percentages indicate the proportion of mothers tested at delivery: N = 376 for stool exams (STH and Giardia); N = 400 for urine exam (for *S*. *haematobium*); N = 437 for antigen/antibody testing (for *Wuchereria* and *S*. *haematobium*)

### Infant vaccine antibody response

Cord blood plasma was available from 573/592 (97%) of the infants followed after birth. In follow up, we were able to examine and test 567 infants at least one time point after birth. Of these, serum was available for 407/567 (72%) of infant subjects at 10-weeks of age, and 440 (78%) at 14-weeks. The 6, 12, 18, and 24-month follow up visits provided data from 462 (81%), 432 (76%), 322 (57%), and 202 (36%) individual infants, respectively. There were only few samples available to analyze from the 30 and 36 month visits, with only 69 (12%) and 9 (1.6%) of the 567 infants represented at these time points.

**Fig 2:**
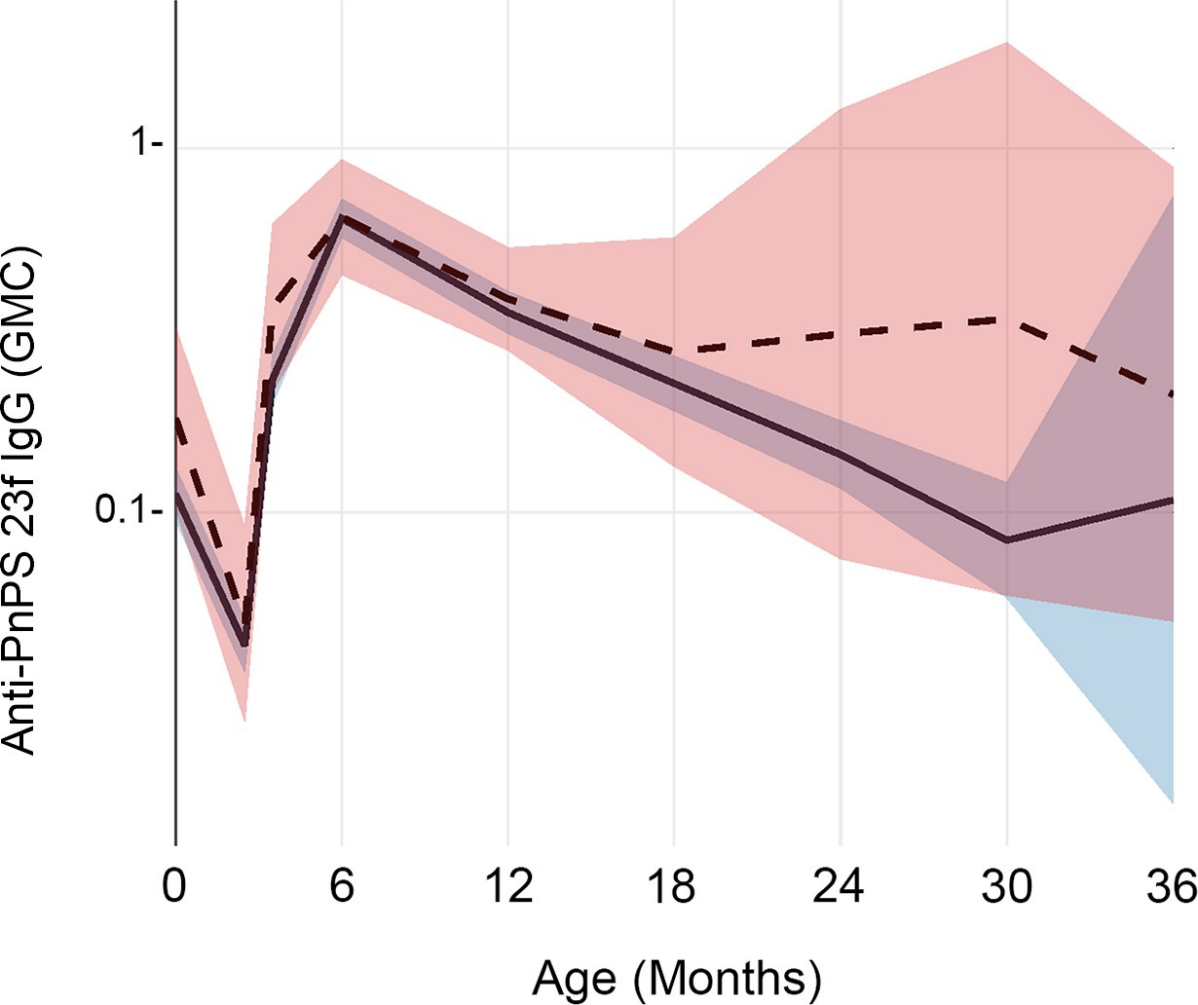
Longitudinal impact of prenatal maternal parasitic infection on infant IgG levels to pneumococcal polysaccharide 23F. This graph shows geometric mean anti-PnPs 23F serum IgG antibody concentrations (in ug/mL) at birth and over the first 36 months of life. The trajectory for children whose mothers had a parasitic infection either during antenatal care or at the time of delivery (infected) is shown by the solid line, with its 95% CI shaded in blue. The trajectory for children whose mothers remained uninfected (uninfected) is shown by the dashed line, with its 95% CI shaded in pink.

In comparing children whose mothers were infected (either during prenatal care or at delivery) to children of uninfected mothers, there was a significant difference in anti-pneumococcal polysaccharide (PnPs) 23F IgG response levels (p = 0.047), as shown in Fig 2. For this antigen, the ‘infected’ group had 0.13 μg/ml less antibody on average compared to the ‘uninfected’ group, with the largest differences noted after the age of 18 months.

**Fig 3:**
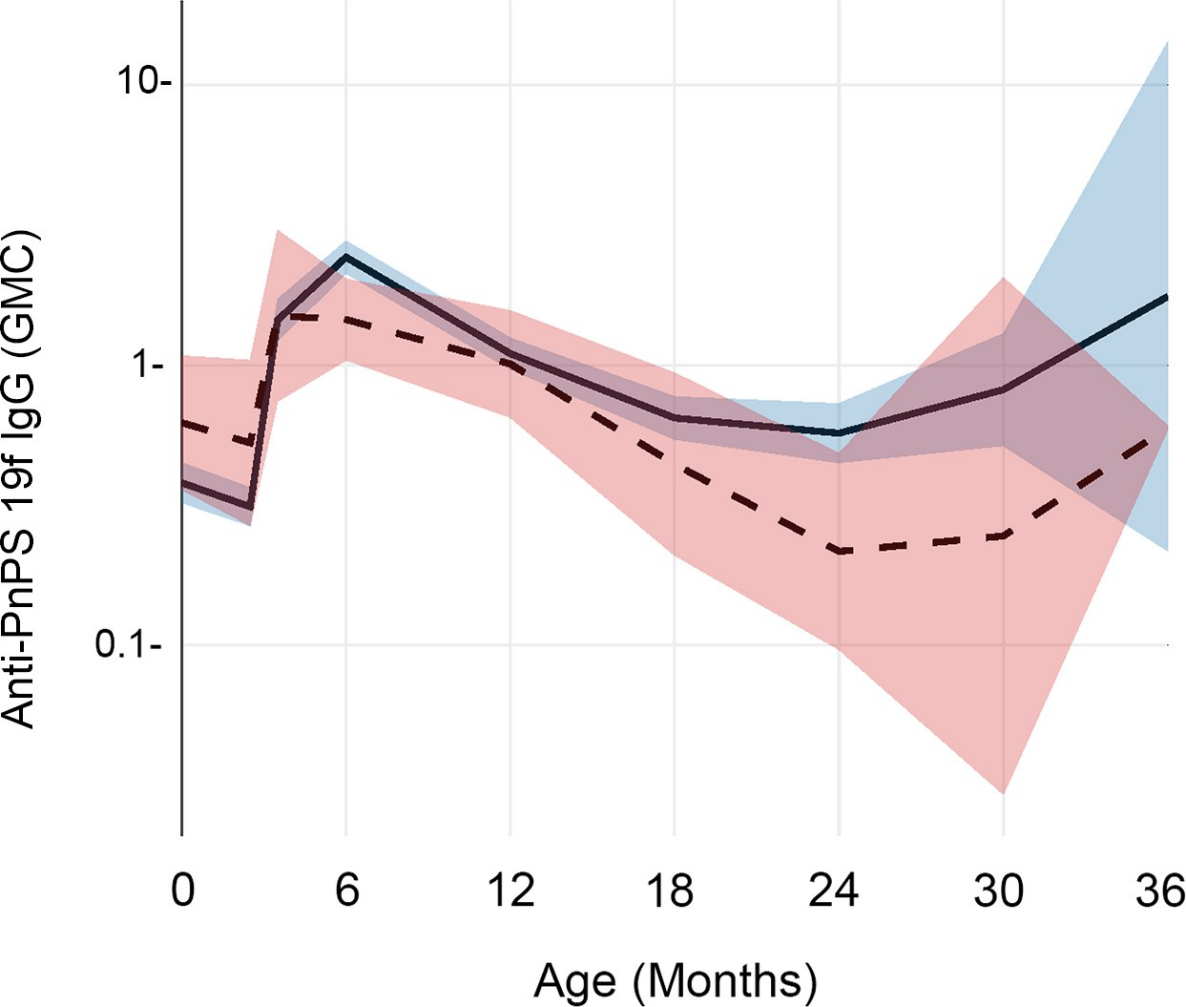
Impact of prenatal maternal parasitic infection on infant IgG levels to pneumococcal polysaccharide 19F. This graph shows geometric mean anti-PnPs 19F serum IgG antibody concentrations (in ug/mL) at birth and over the first 36 months of life. The trajectory for children whose mothers had a parasitic infection either during antenatal care or at the time of delivery (infected) is shown by the solid line, with its 95% CI shaded in blue. The trajectory for children whose mothers remained uninfected (uninfected) is shown by the dashed line, with its 95% CI shaded in pink.

**Fig 4:**
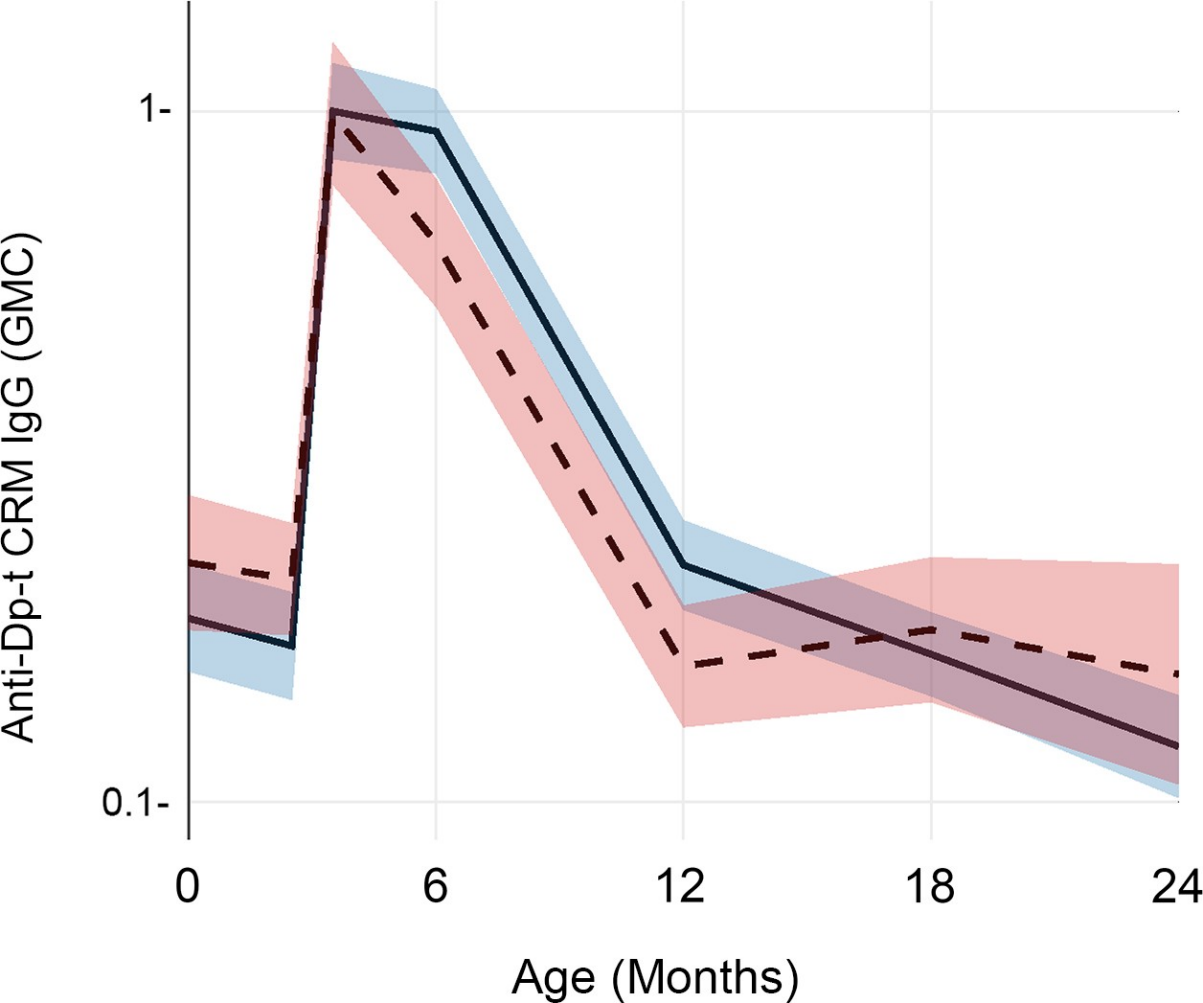
Impact of prenatal maternal parasitic infection on infant IgG levels to diptheria toxoid CRM antigen. This figure shows geometric mean anti-Dp-t_CRM_ serum IgG antibody concentrations (in IU/mL) at birth and over the first 36 months of life. The trajectory for children whose mothers had a parasitic infection either during antenatal care or at the time of delivery (infected) is shown by the solid line, with its 95% CI shaded in blue. The trajectory for children whose mothers remained uninfected (uninfected) is shown by the dashed line, with its 95% CI shaded in pink.

The effect of maternal parasitic infections was not consistent. We found opposite and significant differences between children whose mothers had prenatal or delivery infections and children of uninfected mothers in terms of their anti-PnPs 19F antibody levels, as shown in Fig 3. For this antigen, our linear mixed effects model indicated that the ‘infected’ group had higher anti-PnPs 19F concentration beginning at six months of age (p = 0.007) and continuing until three years of age (p = 0.006). Using the same modeling approach, we found a small but statistically significant (p = 0.001) effect in anti-diphtheria CRM when mothers had infections at delivery, again with higher antibody levels in the children of infected mothers from six to twelve months of age. (Fig 4)

We also examined the separate effects of individual infections and found that many of the more prevalent parasitic infections during pregnancy were associated with minor, mostly enhancing effects on infant anti-vaccine antibody levels after 18–24 months of age. The parasite exposures were malaria, schistosomiasis, filaria, hookworm, and an ‘any soil-transmitted helminth’ category. The magnitude of their effects was typically less than 0.1 μg/ml IgG for any antigen (see S1–S5 Figs for details). In assessing the impact of two or more antenatal maternal infections (‘polyparasitism’), we noted non-significant increases in antibody concentrations in later infancy for antigens PnPs 5, 7F, and 9V among children of infected mothers (S6 Fig). Of note, we did not observe significant differences in post-vaccination IgG levels among children of mothers with heavy (≥ 50 eggs/10 mL urine, N = 15) vs. light (1–49 eggs/10 mL urine, N = 36) vs. no *S*. *haematobium* infection (N = 383). In addition, there were no significant differences in infant responses between those with malaria-parasitemic mothers who did (N = 185) or did not (N = 47) clear their parasitemia during antenatal care. Finally, there were no differences in response between infants whose mothers did (N = 116) or did not (N = 82) clear their documented STH infections during pregnancy.

### Presence or absence of protective levels of circulating antibody

**Fig 5:**
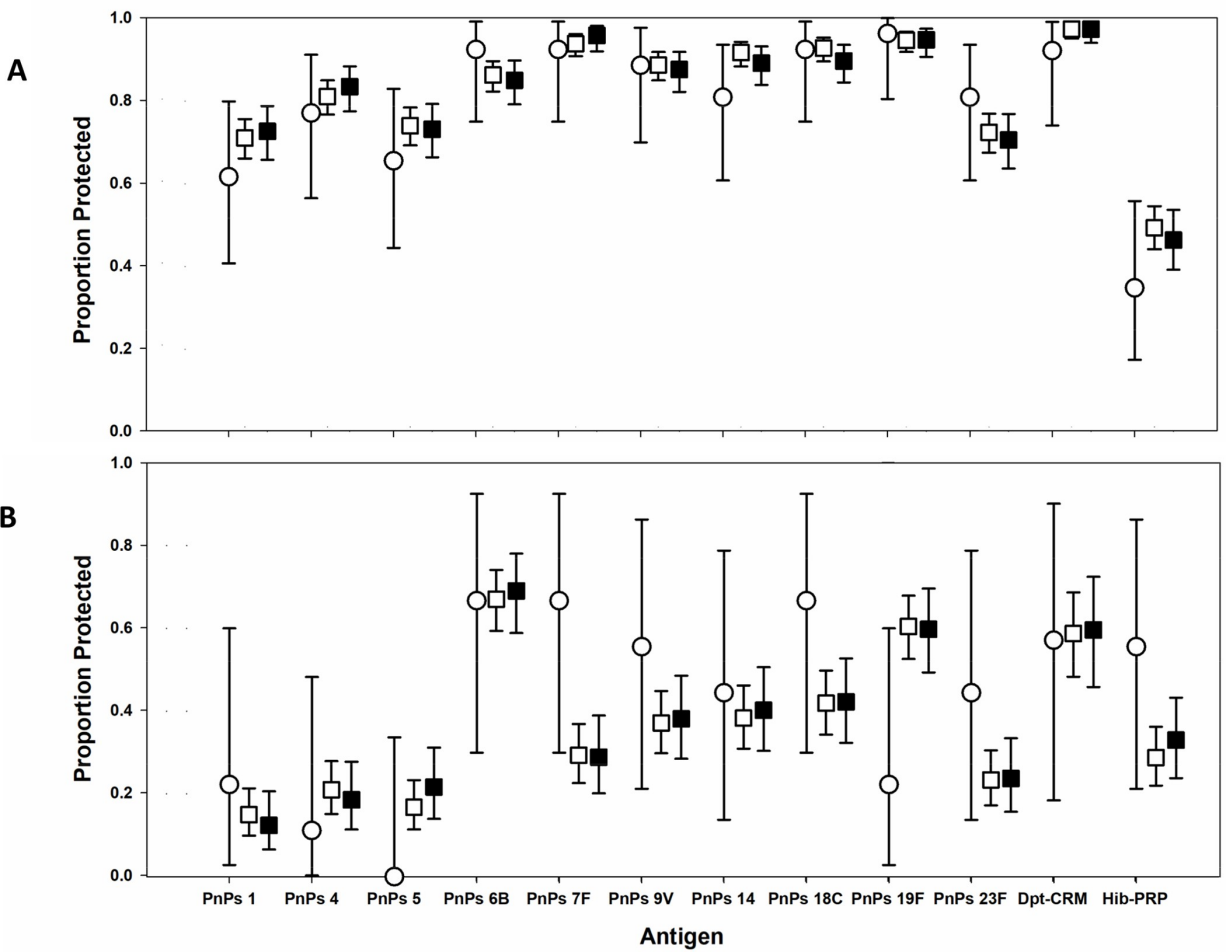
Effect of prenatal maternal parasitic infection on prevalence of protective infant anti-vaccine IgG levels at 6 and 24 months of age. The upper panel (A) indicates results at 6 months of age. The lower panel (B) indicates results at 24 months of age. Open circles indicate the proportion of children of mothers uninfected during pregnancy who had protective antibody levels against each of vaccine antigens tested in this study. Antigens are arrayed along the x axis; error bars indicate the 95% CI of the observed prevalence values. Open boxes indicate results for children of mothers who had any antenatal parasite infection, and black boxes indicate results for children whose mothers had two or more infections during pregnancy. Numerical details are presented in S1 Table.

We next determined the proportion of infants who achieved successful vaccine protection, i.e., who had concentrations of antibody considered protective against invasive disease. (Fig 5, S1 Table). At birth, the uninfected group had a wide range of antibody concentrations against the 12 antigens tested, with rates of protective titers ranging from 3% for PnPs 1 to 83% for PnPs 14. At six months, PnPs 6B, 7F, 9V, 14, 18C, 19F, 23F, and Dp-t-CRM antibodies were at protective levels for >80% of the children whose mothers were uninfected prenatally (range of 81–96%). By 24 months, these rates of protection decreased, with percent protective levels ranging from 0% to 67%, with protective rates <50% for six of the twelve antigens. However, the number of children whose mothers had been uninfected, and who had serum available for testing at this age, was low (S1 Table, N = 9). Patterns of protection for the maternally-infected and–polyparasitized groups were similar (Fig 5). The only significant differences found were lower rates of protection against PnPs 7F and higher rates of protection against PnPs 19F at age 24 months among children of infected mothers (S1 Table).

## Discussion

This prospective cohort study examined the effects of maternal prenatal parasitic infections on later infant vaccination responses to diphteria toxoid and to antigens used in pneumococcal and Hib conjugate vaccines. It found limited influence of prenatal maternal infection status on infant post-vaccination antibody levels. In contrast to the findings in our previous 2006–2009 cohort study [15, 16], most of the current 2013–2015 cohort’s infants with *in utero* exposure to parasitic infections had an increased or equivalent antibody response to nine out of ten pneumococcal antigens, to diphtheria, and to *H*. *influenzae* type B, as compared to children of uninfected mothers. Post-vaccination levels of IgG were significantly lower only for *S*. *pneumoniae* serotype 23F, the most common serotype found among local school age children [28]. Most of the observed differences in IgG levels between exposed and unexposed infants were transient during the first two years of life; when comparing putative protection rates based on threshold antibody levels, prenatally exposed infants were similar to their unexposed peers.

These results are in contrast to previous findings of studies of the negative impact of concurrent or prenatal exposure to parasitic infections on vaccination efficacy, both from our group as well as others [9–11, 14, 17, 18, 29]. This was, however, the first study of the effects of parasitic infections in pregnancy on infant response to the *S*. *pneumoniae* conjugate vaccine. Of note, two other studies have shown increased vaccine antigen responses in infants exposed to maternal parasitic infections. Specifically, following BCG vaccination, IFN-γ response to PPD challenge was higher among infants with prenatal *T*. *cruzi* exposure [30], and plasma vaccine-specific IgA levels after oral polio and rotavirus vaccination was greater in infants of mothers with prenatal helminth infections [31].

An important consideration for the differences in results may be due to the fact that, in the current 2013–2015 cohort, nearly all of the mothers received more aggressive anti-parasitic therapy for malaria and intestinal helminths during pregnancy, whereas this was not the case in the previous 2006–2009 cohort. Because prophylaxis is now standard of care in Kenya, it was not possible to test the differential effect of preventive treatment on the immune response in the infants in a more robust trial design, but intercurrent treatment enhancements may have reversed the detrimental effect of prenatal parasitic infections that we previously observed [15, 16]. It should be noted that certain infections such as *S*. *haematobium* and *Trichuris* would not have been effectively cleared by the anti-parasitic antenatal treatment with mebendazole given to the mothers in this cohort. However, the current, more aggressive IPTp dosing and follow-up treatments for detectable *Plasmodium* and STH infections were important secular changes between our earlier 2006–2009 cohort (which had limited IPTp and albendazole preventive therapy) and the 2012–2015 cohort studied here. In the 2006–2009 cohort, IL-10 immunomodulatory cytokine responses to filaria and to *S*. *haematobium* were significantly associated with reductions in Hib and diphtheria vaccine responses and/or duration of effect [16]. In current 2012–2015 cohort, prenatal prevalence of filariasis among mothers was 23%, lower than the 44% found in the 2006–2009 cohort [15], this effect likely related to a regional filariasis control campaign implemented during intervening years. The lower prevalence of filariasis and the more effective preventive/suppressive therapies used for parasite control may have reduced the impact of parasitic infections on Hib and Dp-t vaccination responses (as observed in 2006–2009 cohort [15, 16]), taking them down to insignificant levels in the current cohort.

Our study classified infection status based on positive testing without respect to clinical status. We used highly sensitive parasite testing for malaria, filaria, and urogenital schistosomiasis, which detects parasitic exposure but does not diagnose clinical disease. Most women in the study were asymptomatic at the time of testing, and it is possible that symptomatic disease would have had a more significant imprinting effect than we observed in our cohort. It is possible that the single stool exams used in our study may have missed very light infections with STH, which may have led to misclassification of infection status. However, there were no heavy STH infections detected in our cohort. In addition, we did not find significant differences between children of mothers who had heavy vs. light intensity *S*. *haematobium* infections, nor did we find differences between children of mothers who did or did not clear their malaria or STH infections with outpatient treatment.

In clear contrast to our earlier 2006–2009 study [15], a randomized, placebo-controlled trial in Uganda investigating the effects of prenatal anti-helminth therapy with albendazole and/or praziquantel on infant responses to the pentavalent diphtheria-tetanus-pertussis-HBV-Hib vaccine found no effect of anti-helminthic treatment (or antenatal malaria or helminth infections) on infant antibody responses to the five antigens at one year of age [32]. A separate study in western Kenya [29] has examined the impact of third trimester *Plasmodium* and/or *S*. *mansoni* infections on later infant immune responses to parasites and to routine vaccinations. They observed no effects on tetanus or diphtheria protection at the 2 year-old follow up, but found a significantly lower level of protection against measles at this age. We agree with these researchers that the impact of antenatal parasitic infections is quite complex, and undoubtedly influenced by the type and severity of infections, the mother’s underlying nutritional and re-exposure status, and the type of vaccine administered (i.e., whether peptide plus adjuvant, polysaccharide conjugate, or live-attenuated). It is even possible that that prenatal exposure to parasites followed by antiparasite treatment may result in enhanced infant vaccine responses (compared to no exposure) to some vaccine antigens, as we found in the current study.

This study was limited in its analysis of antibody responses among the cohort infants due to the low number of mothers documented to be completely free of the parasitic infections of interest. Non-adherence to interval testing and losses to follow-up limited the available sample size for serologic testing. In our statistical model comparing the interaction of infection status on antibody levels at specific time points, the most significant effects were often found in the 30 and 36 month visits, when there were smaller numbers of observations available for analysis. This significance cannot be extrapolated to the groups as a whole, so that overall exposure class findings are less robust, i.e., we are not able to reliably determine if there is a multi-year effect on vaccine antibody levels in parasite-exposed children. We had a much larger sample pool for earlier time points during the infant follow-ups, especially in the 6–24 month period, which is the critical period when maternally-derived passive protection declines. Although Fig 5B suggests that, at 24 months, among children of infected women (i.e., those prenatally-exposed to parasites) there may be lower rates of protection against pneumococcal serotypes 7F, 9V, 18C, and 23 F and against Hib, the low number of unexposed children available for comparison does not allow for any clear statistical inference in this regard. Of note, our statistical analysis, which included multiple outcomes and multiple time points, did not adjust for multiple comparisons, such that some of the apparent differences reported may be due to random variation in outcomes. Future studies involving stratified enrollment based on more sensitive multiplex diagnostics and allowing for longer interval follow-ups will provide better power to answer this question.

In summary, this study yields additional longitudinal information on the relative impact that fetal parasite exposures may have on vaccine efficacy and early immunity to encapsulated bacterial pathogens. As the global health community moves forward to provide safe and effective vaccinations for *S*. *pneumoniae*, diphtheria, and *H*. *influenzae* [3], it remains more important that we continue to refine our understanding of the ways in which these vaccinations are best administered. Because parasitic infections alter the expected immune response to vaccination in a number of settings [9–13, 15, 16, 29], and because the regions that bear the brunt of mortality from encapsulated bacteria also carry the highest prevalence of parasitic infections, a more in depth knowledge of parasite-vaccine interactions is clearly needed. In the meantime, efforts to assure that adolescent and adult women are included in STH, filariasis, and schistosomiasis control programs should be strengthened, as this group is often wrongly excluded from preventive treatment because of fears about possible adverse drug effects in pregnancy. However, the safety and efficacy of treatment during pregnancy has now been established in placebo-controlled randomized trials [33, 34], and expanded treatments for all women should contribute to global improvement in maternal health, as now prioritized by World Health Organization guidelines [35].

## Supporting information

S1 Fig Effects of maternal prenatal malaria infection status on infant vaccine responses to *S*. *pneumoniae* antigens PnPs 4 and 5, and diptheria CRM. Geometric mean anti-antigen serum IgG antibody concentrations (in ug/mL for PnPS 4 and 5, in IU/ml for diphtheria CRM) at birth and over the first 36 months of life. The trajectory for children whose mothers had malaria either during antenatal care or at the time of delivery (infected) is shown by the solid line, with its 95% CI shaded in blue. The trajectory for children whose mothers remained uninfected (uninfected) is shown by the dashed line, with its 95% CI shaded in pink.
(PDF)

S2 Fig Effects of maternal prenatal hookworm infection status on infant vaccine responses to *S*. *pneumoniae* antigen PnPs 14. Geometric mean anti-antigen serum IgG antibody concentrations (in ug/mL) at birth and over the first 36 months of life. The trajectory for children whose mothers had hookworm either during antenatal care or at the time of delivery (infected) is shown by the solid line, with its 95% CI shaded in blue. The trajectory for children whose mothers remained uninfected (uninfected) is shown by the dashed line, with its 95% CI shaded in pink.
(PDF)

S3 Fig Effects of maternal prenatal *S*. *haematobium* infection status on infant vaccine responses to *H*. *influenzae* PRP antigen. Geometric mean anti-antigen serum IgG antibody concentrations (in ug/mL) at birth and over the first 36 months of life. The trajectory for children whose mothers had *S*. *haematobium* detected either during antenatal care or at the time of delivery (infected) is shown by the solid line, with its 95% CI shaded in blue. The trajectory for children whose mothers remained uninfected (uninfected) is shown by the dashed line, with its 95% CI shaded in pink.
(PDF)

S4 Fig Effects of maternal prenatal filarial infection status on infant vaccine responses to *S*. *pneumoniae* antigen PnPs 5 and to *H*. *influenzae* PRP antigen. Geometric mean anti-antigen serum IgG antibody concentrations (in ug/mL) at birth and over the first 36 months of life. The trajectory for children whose mothers had circulating Og4C3 filarial antigen detected either during antenatal care or at the time of delivery (infected) is shown by the solid line, with its 95% CI shaded in blue. The trajectory for children whose mothers remained uninfected (uninfected) is shown by the dashed line, with its 95% CI shaded in pink.
(PDF)

S5 Fig Effects of maternal prenatal soil-transmitted helminth infection status on infant vaccine responses to *S*. *pneumoniae* antigens PnPs 14, 18C, and 19F, and to diptheria CRM. Geometric mean anti-antigen serum IgG antibody concentrations (in ug/mL for all PnPS, in IU/ml for diphtheria CRM) at birth and over the first 36 months of life. The trajectory for children whose mothers had any soil-transmitted helminth (STH) infection detected either during antenatal care or at the time of delivery (infected) is shown by the solid line, with its 95% CI shaded in blue. The trajectory for children whose mothers remained uninfected (uninfected) is shown by the dashed line, with its 95% CI shaded in pink.
(PDF)

S6 Fig Effects of multiple maternal prenatal parasite infections on infant vaccine responses to *S*. *pneumoniae* antigens PnPs 5, 7F, and 9V. Geometric mean anti-antigen serum IgG antibody concentrations (in ug/mL) at birth and over the first 36 months of life. The trajectory for children whose mothers had multiple parasitic infections detected either during antenatal care or at the time of delivery (infected) is shown by the solid line, with its 95% CI shaded in blue. The trajectory for children whose mothers remained uninfected (uninfected) is shown by the dashed line, with its 95% CI shaded in pink.
(PDF)

S1 Table Proportion of cohort children having protective levels of anti-vaccine antigen IgGs at delivery, and at 6 and 24 months of age. (DOCX)
