# Supplementary figures and images for: Parasitic infections during pregnancy need not affect infant antibody responses to early vaccination against Streptococcus pneumoniae, diphtheria, or Haemophilus influenzae type B
Source: PLoS Negl Trop Dis. 2019 Feb 28;13(2):e0007172. doi: 10.1371/journal.pntd.0007172 (PMC6413956; doi:10.1371/journal.pntd.0007172)

*S. pneumoniae* 14 IgG by hookworm infection status

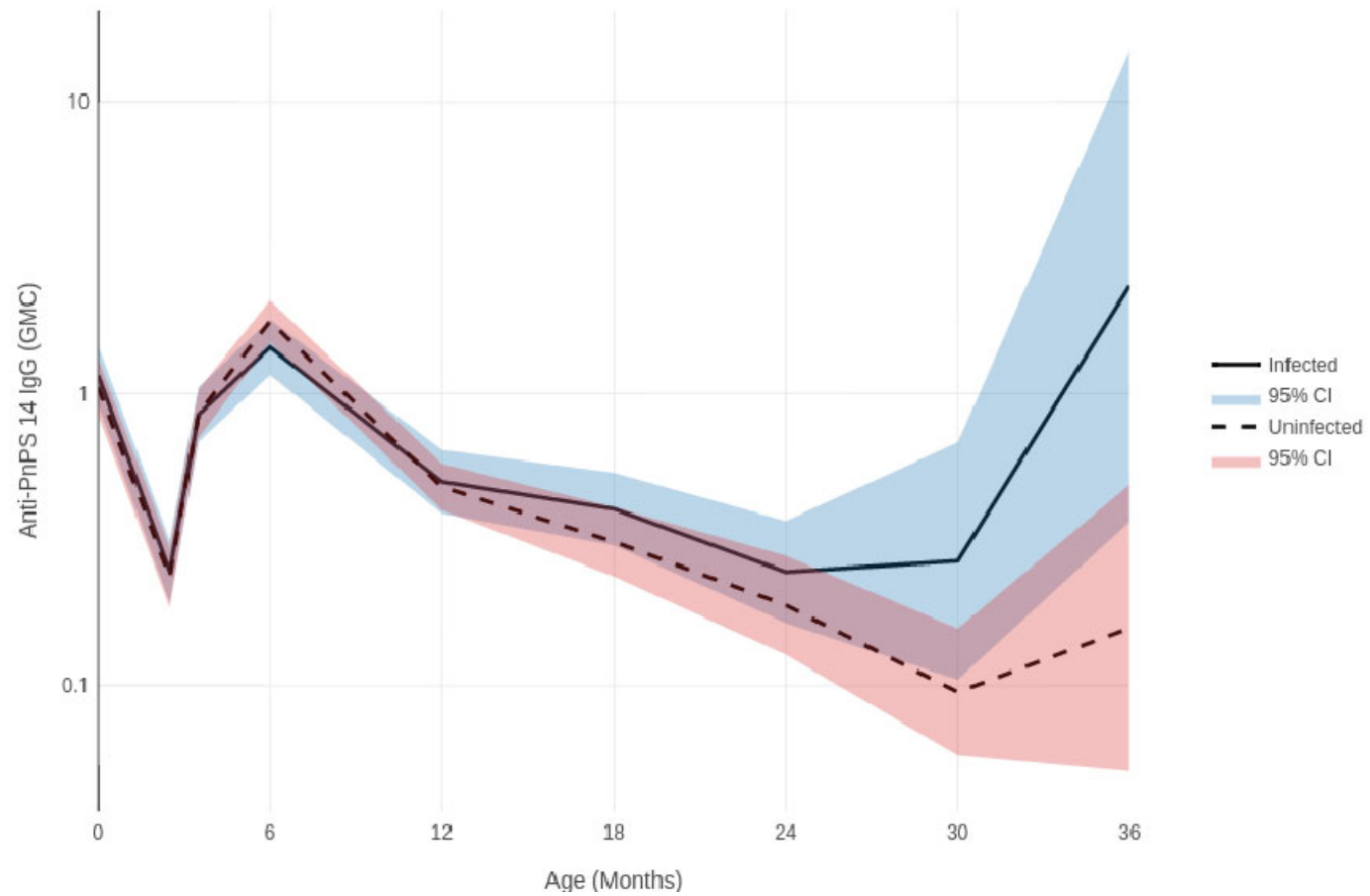

Supplement: S2 Fig — Geometric mean anti-antigen serum IgG antibody concentrations (in ug/mL) at birth and over the first 36 months of life. The trajectory for children whose mothers had hookworm either during antenatal care or at the time of delivery (infected) is shown by the solid line, with its 95% CI shaded in blue. The trajectory for children whose mothers remained uninfected (uninfected) is shown by the dashed line, with its 95% CI shaded in pink. (PDF) [file pntd.0007172.s002.pdf]

*H. influenzae* PRP by *Schistosoma* infection status

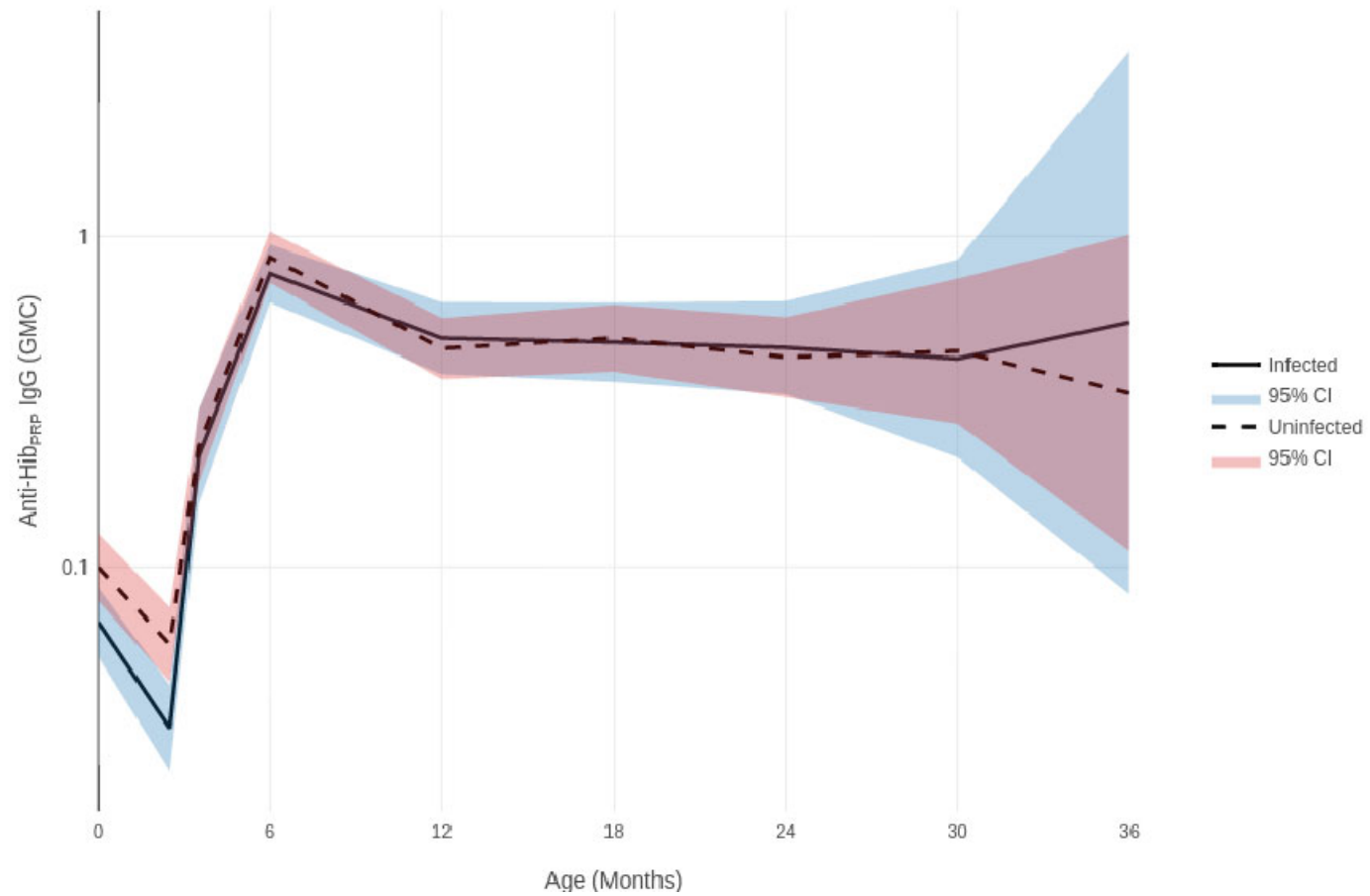

Supplement: S3 Fig — Geometric mean anti-antigen serum IgG antibody concentrations (in ug/mL) at birth and over the first 36 months of life. The trajectory for children whose mothers had S. haematobium detected either during antenatal care or at the time of delivery (infected) is shown by the solid line, with its 95% CI shaded in blue. The trajectory for children whose mothers remained uninfected (uninfected) is shown by the dashed line, with its 95% CI shaded in pink. (PDF) [file pntd.0007172.s003.pdf]
