# Supplementary material for: Parasitic infections during pregnancy need not affect infant antibody responses to early vaccination against Streptococcus pneumoniae, diphtheria, or Haemophilus influenzae type B
Source: PLoS Negl Trop Dis. 2019 Feb 28;13(2):e0007172. doi: 10.1371/journal.pntd.0007172 (PMC6413956; doi:10.1371/journal.pntd.0007172)

(a) *S. pneumoniae* 14 IgG by STH infection status

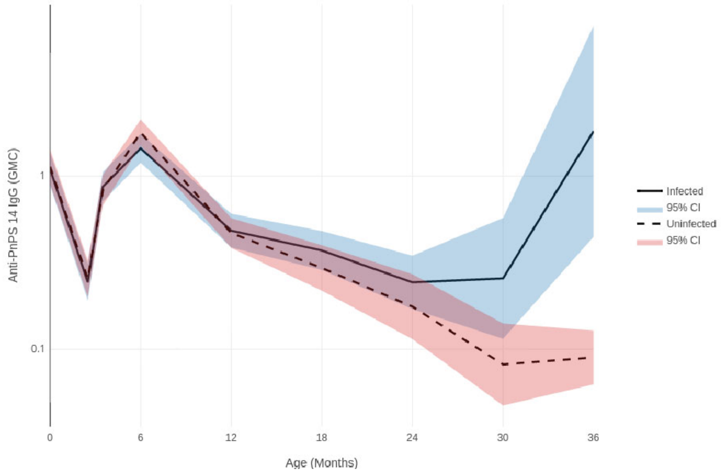

(b) *S. pneumoniae* 18C IgG by STH infection status

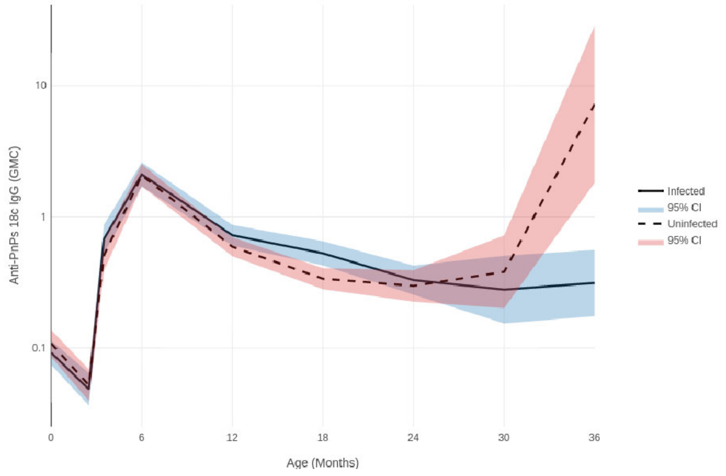

(c) *S. pneumoniae* 19F IgG by STH infection status

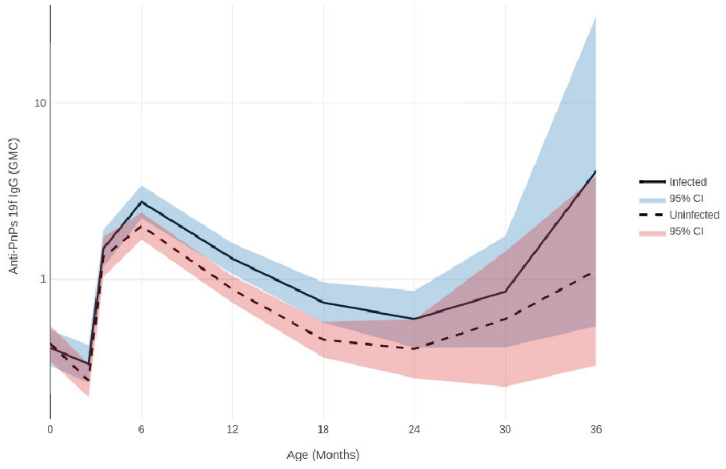

(d) Diphtheria CRM IgG by STH infection status

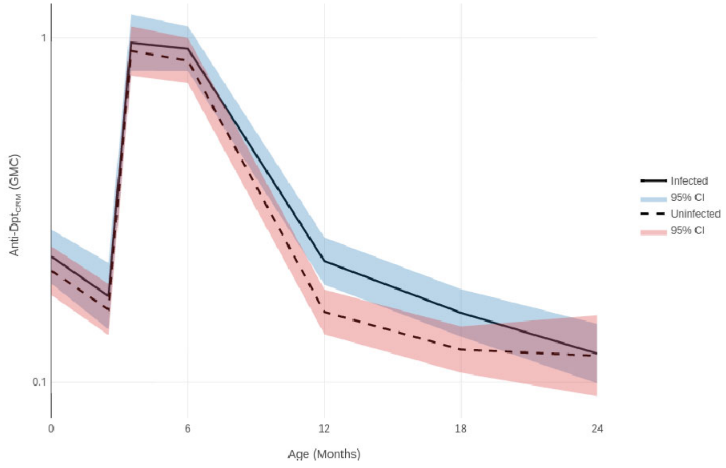

Supplement: S5 Fig — Geometric mean anti-antigen serum IgG antibody concentrations (in ug/mL for all PnPS, in IU/ml for diphtheria CRM) at birth and over the first 36 months of life. The trajectory for children whose mothers had any soil-transmitted helminth (STH) infection detected either during antenatal care or at the time of delivery (infected) is shown by the solid line, with its 95% CI shaded in blue. The trajectory for children whose mothers remained uninfected (uninfected) is shown by the dashed line, with its 95% CI shaded in pink. (PDF) [file pntd.0007172.s005.pdf]
