# Supplementary material for: Parasitic infections during pregnancy need not affect infant antibody responses to early vaccination against Streptococcus pneumoniae, diphtheria, or Haemophilus influenzae type B
Source: PLoS Negl Trop Dis. 2019 Feb 28;13(2):e0007172. doi: 10.1371/journal.pntd.0007172 (PMC6413956; doi:10.1371/journal.pntd.0007172)
Supplement: S1 Table — (DOCX) [file pntd.0007172.s007.docx]

**Table S1. Proportion of cohort children having protective levels of anti-vaccine antigen IgGs at delivery, 6, and 24 mos of age**

|  | **At delivery (cord blood)** | | | | **At six months** | | | | **At 24 months** | | | |
| --- | --- | --- | --- | --- | --- | --- | --- | --- | --- | --- | --- | --- |
| **Antigen^a^** | **Mother uninfected (N=35)** | **Mother infected (N=462)** | **Mother with ≥ 2 infections**  **(N=252)** | ***P* value^c^** | **Mother uninfected (N=26)** | **Mother infected (N=366)** | **Mother with ≥ 2 infections (N=194)** | ***P* value^c^** | **Mother uninfected (N=9)** | **Mother infected (N=167)** | **Mother with ≥ 2 infections (N=97)** | ***P* value^c^** |
| **PnPs 1** | 1 (3%) | 16 (3%) | 7 (3%) | 1.0 | 16 (62%) | 258 (71%) | 141 (73%) | 0.38 | 2 (22%) | 25 (15%) | 12 (12%) | 0.63 |
| **PnPs 4** | 7 (20%) | 56 (12%) | 26 (10%) | 0.19 | 20 (77%) | 295 (81%) | 162 (84%) | 0.61 | 1 (11%) | 35 (21%) | 18 (19%) | 0.69 |
| **PnPs 5** | 3 (9%) | 19 (4%) | 9 (4%) | 0.20 | 17 (65%) | 269 (74%) | 142 (73%) | 0.37 | 0 (0%) | 28 (17%) | 21 (22%) | 0.36 |
| **PnPs 6B** | 11 (31%) | 98 (21%) | 55 (22%) | 0.20 | 24 (92%) | 314 (86%) | 165 (85%) | 0.56 | 6 (67%) | 112 (67%) | 67 (69%) | 1.0 |
| **PnPs 7F** | 6 (17%) | 94 (20%) | 54 (21%) | 0.83 | 24 (92%) | 342 (93%) | 186 (96%) | 0.69 | **6 (67%)** | **49 (29%)** | 28 (29%) | **0.028** |
| **PnPs 9V** | 8 (23%) | 71 (15%) | 42 (17%) | 0.24 | 23 (88%) | 323 (88%) | 170 (88%) | 1.0 | 5 (56%) | 63 (37%) | 37 (38%) | 0.31 |
| **PnPs 14** | 29 (83%) | 361 (78%) | 203 (81%) | 0.67 | 21 (81%) | 334 (91%) | 173 (89%) | 0.09 | 4 (44%) | 64 (38%) | 39 (40%) | 0.74 |
| **PnPs 18C** | **13 (37%)** | **91 (20%)** | 50 (20%) | **0.03** | 24 (92%) | 338 (92%) | 174 (90%) | 1.0 | 6 (67%) | 70 (42%) | 41 (42%) | 0.18 |
| **PnPs 19F** | 23 (66%) | 248 (54%) | 135 (54%) | 0.22 | 25 (96%) | 345 (94%) | 184 (95%) | 1.0 | **2 (22%)** | **101 (60%)** | 58 (60%) | **0.035** |
| **PnPs 23F** | 12 (34%) | 134 (29%) | 77 (31%) | 0.56 | 21 (81%) | 263 (72%) | 137 (71%) | 0.37 | 4 (44%) | 39 (23%) | 23 (24%) | 0.23 |
| **Dpt-CRM^b^** | 23 (66%) | 333 (73%) | 189 (75%) | 0.43 | 23 (92%) | 353 (97%) | 188 (97%) | 0.20 | 4 (57%) | 57 (59%) | 34 (60%) | 1.0 |
| **Hib-PRP** | 6 (17%) | 37 (8%) | 20 (8%) | 0.11 | 9 (35%) | 178 (49%) | 90 (46%) | 0.22 | 5 (56%) | 48 (29%) | 32 (33%) | 0.13 |

^a^ Abbreviations: PnPS, pneumococcal polysaccharide; Dpt-CRM, diptheria toxoid; Hib-PRP, *H. influenzae* B polyribitol

phosphate.

^b^ Due to assay failure, not all samples were successfully tested for anti-Dpt-CRM: **at delivery**, infeccted N= 458, ≥2 infections N=251; **at 6 months**, uninfected N=25, infected N=364, ≥2 infections N=193; **at 24 months**, uninfected N=7, infected N=97, ≥2 infections N=57 for this one assay.

^c^ P values from comparison of rates by two-tailed Fisher’s exact test for chldren of maternally-infected vs. maternally-uninfected groups. Significant differences are indicated in **bold** font.
